# Advancing crop disease resistance through genome editing: a promising approach for enhancing agricultural production

**DOI:** 10.3389/fgeed.2024.1399051

**Published:** 2024-06-26

**Authors:** Subaya Manzoor, Sajad Un Nabi, Tariq Rasool Rather, Gousia Gani, Zahoor Ahmad Mir, Ab Waheed Wani, Sajad Ali, Anshika Tyagi, Nazia Manzar

**Affiliations:** ^1^ Division of Plant Pathology, FOA-SKUAST-K, Wadura, Srinagar, India; ^2^ ICAR-Central Institute of Temperate Horticulture, Srinagar, India; ^3^ Division of Plant Pathology, SKUAST-K Shalimar, Srinagar, India; ^4^ Division of Basic Science and Humanities, FOA-SKUAST-K, Wadura, Srinagar, India; ^5^ Department of Plant Science and Agriculture, University of Manitoba, Winnipeg, MB, Canada; ^6^ Department of Horticulture, LPU, Jalander, Punjab, India; ^7^ Department of Biotechnology, Yeungnam University, Gyeongsan, Republic of Korea; ^8^ Plant Pathology Lab, ICAR-National Bureau of Agriculturally Important Microorganism, Mau, Uttar Pradesh, India

**Keywords:** pathogens, disease resistance, genome editing crispr, meganucleases, TALEN, ZFN

## Abstract

Modern agriculture has encountered several challenges in achieving constant yield stability especially due to disease outbreaks and lack of long-term disease-resistant crop cultivars. In the past, disease outbreaks in economically important crops had a major impact on food security and the economy. On the other hand climate-driven emergence of new pathovars or changes in their host specificity further poses a serious threat to sustainable agriculture. At present, chemical-based control strategies are frequently used to control microbial pathogens and pests, but they have detrimental impact on the environment and also resulted in the development of resistant phyto-pathogens. As a replacement, cultivating engineered disease-resistant crops can help to minimize the negative impact of regular pesticides on agriculture and the environment. Although traditional breeding and genetic engineering have been instrumental in crop disease improvement but they have certain limitations such as labour intensity, time consumption, and low efficiency. In this regard, genome editing has emerged as one of the potential tools for improving disease resistance in crops by targeting multiple traits with more accuracy and efficiency. For instance, genome editing techniques, such as CRISPR/Cas9, CRISPR/Cas13, base editing, TALENs, ZFNs, and meganucleases, have proved successful in improving disease resistance in crops through targeted mutagenesis, gene knockouts, knockdowns, modifications, and activation of target genes. CRISPR/Cas9 is unique among these techniques because of its remarkable efficacy, low risk of off-target repercussions, and ease of use. Some primary targets for developing CRISPR-mediated disease-resistant crops are host-susceptibility genes (the S gene method), resistance genes (R genes) and pathogen genetic material that prevents their development, broad-spectrum disease resistance. The use of genome editing methods has the potential to notably ameliorate crop disease resistance and transform agricultural practices in the future. This review highlights the impact of phyto-pathogens on agricultural productivity. Next, we discussed the tools for improving disease resistance while focusing on genome editing. We provided an update on the accomplishments of genome editing, and its potential to improve crop disease resistance against bacterial, fungal and viral pathogens in different crop systems. Finally, we highlighted the future challenges of genome editing in different crop systems for enhancing disease resistance.

## Introduction

Microbial pathogens are a constant threat to agricultural productivity, economy and food security (Manzoor et al., 2023). Furthermore, climate change not only affects the plant defence system but also increases the rate of plant disease outbreaks, their distribution and host specificity, thereby putting the global food supply and ecological biodiversity at risk (Bebber, 2015) primary goal of contemporary agriculture is to secure long-term food security while also preserving environmental sustainability. As the world’s population has continually increased in recent years, food security has emerged as a serious global concern. It has become increasingly important to consider the problem of feeding the world’s expanding population of billions while preserving the environment’s balance (Pandit et al., 2022). However, phytopathogens, which represent a danger to the sustainability of food globally, limit the yield of crops. Plant pests and pathogens lead to yield losses globally in rice up-to 30.3%, wheat (30.3%), maize (22.6%) and potato (17.2%) (Savary et al., 2012). Fungal diseases alone in cereals result in yield losses of about (15%–2%) and in extreme cases upto 60% (Rozewicz et al., 2021). Phoma blight in soybeans leads to a yield loss of 51.72% (Manzoor et al., 2023). *Fusarium* root rot in field peas leads to a yield loss of about 60% (Gossen et al., 2016). The agriculture sector mainly relies on chemical control methods to battle infections in the absence of genetic resistance. However, the use of pesticides and other chemical agents presents issues with the direct or indirect safety of other living things (Damalas et al., 2011). Repeated use of chemicals has polluted the water, air and soil ecosystems, thereby bioaccumulating at higher tropic levels. About 0.1% of chemicals reach the target pathogens, rest accumulate in the surrounding environment (Gill and Garg, 2014). Considering their extensive and sporadic application, phytopathogens have still grown more resilient to several chemical treatments (Ahmad et al., 2020). *Colletotrichum truncatum* which causes soybean anthracnose, has developed resistance against fungicides such as carbendazim, difenoconozole, azoxystrobin and penthiopyrad. Pathogens have undergone mutations in the beta–tubulin gene, the cytochrome b gene, and the gene encoding the succinate dehydrogenase subunit, which were used as target sites for fungicides (Poti et al., 2023). Reducing the reliance on chemical control in food production has become a critical goal to address the considerable impact of global climate change (Pachauri & Reisinger, A, 2007) and minimize the detrimental environmental effects connected with existing practices (Tilman et al., 2002). Crops with better qualities and performance have been made possible by the processes of plant domestication and breeding. The production of resistant plant types has the most promise for the sustainable control of plant diseases, providing advantages on the social, economic, and environmental fronts. To supply the nutritional needs of human populations, genetic resources that provide resistance to pests and diseases are crucial resources for crop improvement (Mundt, 1994). Traditional breeding techniques have historically been used in conventional resistance breeding to introduce naturally occurring or artificially generated mutant alleles into desired genotypes. Although these genetic methods of disease control have shown significant results over many years, they also have several drawbacks. Notably, such techniques can only be used on plants with adequate genetic diversity and crossbreeding propensity. Additionally, because these methods might transfer several characteristics, covering broad genomic areas rather than only the intended resistant trait (single gene insertion), they may be inaccurate and ambiguous. Additionally, the selection of progeny and the process of genetic crossover can be labour- and time-intensive. Also, most contemporary cultivars that have been chosen to increase yield values are comparatively more vulnerable to infections. Conventional practices must alter to handle the problems caused by the dynamic global food demand landscape, developing diseases, and the period of climate change. The availability of genome and transcriptome sequences requires the adaptation of standard methods (Gao, 2018). The following disadvantages of conventional resistance breeding are present: The first is how time- and labour-intensive it is to produce resistant cultivars. The second phenomenon is linkage drag, wherein top cultivars unintentionally acquire unfavourable traits along with the resistant gene. Third, traditional breeding is only applicable to crossable genotypes. An alternate strategy is mutation breeding, which involves inducing changes in the plant’s DNA using chemical or physical mutagenesis. This technique produces mutants, which are then subjected to stringent selection to assess their desirable traits. This approach also helps with the discovery and mapping of new genes in the genome. However, mutant breeding is a laborious and time-consuming procedure that normally takes 6–7 years to produce results. The existence of random mutations in the genome, which can occasionally be hard to recognize and anticipate, is a significant drawback of this method (Ahmad et al., 2020). Transgenic technology, in particular genetically modified (GM) crops, has emerged as a viable remedy in light of the difficulties faced by agricultural scientists and farmers. Transgenic technology is a flexible method that allows the inclusion of genes from many sources and is not restricted to crossable genotypes. Utilizing this technique, scientists have created crops with much higher yields, increased resilience to biotic and abiotic stressors, herbicide resistance, and greater nutritional value. Regulatory committees have highlighted considerable acceptability problems for transgenic crops, notwithstanding these benefits (Gao, 2021).

In recent years, the advancement in genome editing approach has revolutionized the plant biology and made it possible to precisely modify crops’ genetic makeup to produce desired features. The term “genome editing” refers to a range of technologies that enable the insertion, deletion, or modification of genetic information at certain sites within the genome of a living creature. The term “new breeding techniques” (NBT) refers to modern, precise molecular methods for focusing on one or more genes. The mechanism of DNA double-strand break (DSB) repair, in which sequence-particular nucleases recognize certain DNA sequences, is the basis for the core processing in genome editing. Nonhomologous end joining (NHEJ) and homologous recombination (HR) are the two main mechanisms by which DSBs are repaired (Voytas and Gao, 2014). Using NHEJ or HR to precisely alter genes, new plant types with favourable agronomic features may be created. While HR uses homologous sequences as templates to recreate the lacking DNA sequences at the breakpoint, NHEJ uses distinct enzymes for DSB repair. Even though NHEJ is error-prone and can result in insertion or deletion alterations, the majority of cells frequently utilize it for DSB repair. Although when a donor DNA template is present, the HR route predominates, leading to precise and targeted alterations. Genome editing uses a variety of sequence-specific nucleases, including base editing, meganucleases (MNs), transcription activator-like effector nucleases (TALENs), zinc finger nucleases (ZFNs), clustered regularly interspaced short palindromic repeats (CRISPR), and CRISPR-associated protein (Cas9). These genome editing tools provide democratic approaches and are distinguished by their quick and low-cost manufacture, making them available to public-private partnerships with nonprofit objectives in addition to private businesses and multinational corporations (Ricroch, 2019). They are already used in many public laboratories. Gene editing tools like CRISPR/Cas9, which are especially efficient and practical, function without the use of proteins or engineering processes. Cas9 nucleases target DNA with single-guide RNA (sgRNA), which is formed from the duplex-RNA structure incorporated with CRISPR RNA (crRNA) and trans-activating crRNA (tracrRNA). The method effectively targets certain genomic regions by changing the dual crRNA-tracrRNA structure into a single-guide RNA (Ahmad et al., 2020). The range of its uses has also been broadened by recent developments in CRISPR-Cas13 technology, base editors, and prime editors (Molla et al., 2020).

The growing abundance of recent studies on plant genome editing through GE technology suggests its practicality, owing to its heightened success rate and user-friendly nature. Beyond plant applications, CRISPR/Cas9 has been employed to target genes encoding proteins involved in interactions between host plants and fungal or oomycete pathogens. This facilitates the exploration of the molecular mechanisms underlying host-pathogen recognition and enables the development of screening systems for disease resistance (Fang and Tyler, 2016). In this review, we highlighted an overview of genome editing technologies and their applications in bolstering plant disease resistance. We delve into the mechanisms underlying genome editing and explore recent advancements in CRISPR/Cas-based tools, including base editing and prime editing. Furthermore, we investigate the potential impact of genome editing on sustainable farming practices, global food security and shaping the future of agriculture. Finally, we highlight the challenges and ethical concerns associated with the widespread adoption of genome-edited crops and propose potential strategies to address these issues. By highlighting recent developments and prospects in genome editing for plant disease resistance, this review aims to contribute to a deeper understanding of this innovative approach and its implications for agriculture and society.

## Impact of various fungal, viral, bacterial diseases on economically important crops

Phytopathogens pose a significant threat to the agricultural economy, causing up to a 40% yearly production loss in economically important crops (Baldi and La Porta, 2020). The financial impact of these losses is substantial, amounting to an estimated $220 billion each year (FAO, 2019). The issue is intensified by the surge in global commerce, which facilitates the fast spread of invasive pathogens, resulting in significant crop damage and reduced yields (Baldi and La Porta, 2020). For instance, numerous soil-borne diseases, including plant-parasitic nematodes, *Fusarium* species, *Rhizoctonia* species, and Pythium species, can cause major production losses in legume crops, whether they occur alone or in combination (Panth et al., 2020). Similar to rice, *Bipolaris oryzae*, a dematiaceous hyphomycete, is responsible for the devastating brown spot diseases that affect rice. This contributed to the 1943 Bengal famine in India. The same fungus is still present in important rice-growing regions of the world, despite the fact that it has not recently produced any significant outbreaks (Sobanbabu et al., 2018). Worldwide crop losses and starvation have been attributed to graminicolous hyphomycetes, which are linked to cereal crops and their wild cousins. These phytopathogens have been shown to be highly destructive. Southern corn leaf blight, which is caused by *Bipolaris maydis*, has been linked to significant crop losses worldwide (Manzar et al., 2022) and *Exserohilum turcicum,* which causes northern corn leaf blight. Another typical leaf spot pathogen in barley and wheat is *Bipolaris sorokiniana* (Kashyap et al., 2023). *Pyricularia oryzae*, also known as *Magnaporthe oryzae,* is a fungal disease that causes frequent outbreaks and is thought to be the most devastating pathogen in rice cultivars. Up to 30% of the world’s rice harvest is lost to this disease every year, making it difficult to control and potentially causing humanitarian and economic problems, especially in Asia (Savary et al., 2019). Many studies on the molecular causes of illnesses and host-pathogen interactions use *P. oryzae*. Another invasive disease that started in the United Kingdom in 1994 is boxwood blight, which is now found in Europe, Asia, New Zealand, and North America (LeBlanc et al., 2018). Boxwood blight, as its name implies, is a disease that affects boxwood (*Buxus* spp.) and results in latent dieback of the leaves as well as fast defoliation (LeBlanc et al., 2018). Similarly, the growth and yield of boxwood in nurseries and landscape plantings are at risk due to recent outbreaks of boxwood blight disease, which is brought on by the fungus *Calonectria pseudonaviculata*. This poses a serious danger to the ornamental plant sector (LeBlanc et al., 2018). After contracting *C. pseudonaviculata*, plants become debilitated, which can lead to plant stress and secondary invader colonization, which frequently results in plant mortality. A wide variety of crops and non-crop plants are affected by foliar anthracnose, which is typically found in tropical and subtropical regions (LeBlanc et al., 2018). As a result, foliar *Colletotrichum* species are regarded as a major source of pre- and post-harvest loss of a wide range of high-value crops, and they are also regularly discovered in plant biosecurity interceptions. Apple mosaic virus (ApMV) and a recently reported novel virus, apple necrotic mosaic virus (ApNMV), linked to mosaic disease in apples, represent a significant economic threat to the apple industry (Nabi et al., 2020). Viral infections affect the entire plant system, are systemic, and are capable of affecting the overall health of orchards (Manzoor et al., 2023; Nabi et al., 2023). The Geminiviridae single-stranded DNA viruses are represented by two viruses: tomato yellow leaf curl virus (TYLCV) and african cassava mosaic virus (ACMV), both of which have enormous economic importance and cause huge economic losses, much of which is exacerbated by efficient transmission via whitefly vectors. The yearly losses from ACMV (and related species) are currently estimated to be between $1.9 and $2.7 billion, with the cassava disease epidemic in East and Central Africa generating significant misery and concerns.

The yield loss estimated due to various diseases is illustrated in (Table 1). Addressing these challenges is critical, and one potential approach is the use of genome editing technology in disease management. Genome editing has emerged as a cutting-edge technique with the potential to revolutionize the way we combat plant diseases. Researchers can increase plant resilience to pathogens by accurately changing their genetic composition, lowering susceptibility and output losses. This novel methodology offers a focused and effective method for creating crops with better disease resistance. Scientists may use genome editing tools like CRISPR-Cas9 to make precise changes to particular genes linked with disease susceptibility, therefore improving the plant’s natural defensive systems (Bi et al., 2024). The use of genome editing in agriculture holds promise for developing resilient crops that can survive the encounters posed by emerging diseases. As we strive to ensure global food production and mitigate the economic effects of plant diseases, genome editing emerges as a potent tool in the goal of sustainable and resilient agriculture. By harnessing the potential of genetic modification, we may aim to reduce the destructive consequences of plant diseases while also ensuring a more secure and productive future for agriculture.

**TABLE 1:** Impact of foliar, soil borne pathogenic fungal, bacterial and viral diseases on crop productivity.

Host	Foliar pathogens	Disease	Yield losses (%)	References
*Zea mays* (maize)	*Exserohilum turcicum*	Northern leaf blight of corn	40–70	Hernandez-Restrepo et al. (2018)
*Z. mays* (maize)	*E. turcicum*	Northern leaf blight of corn	40–70	Hernandez-Restrepo et al. (2018)
*Z. mays* (maize)	*Bipolaris maydis*	Southern leaf blight of corn	30 to 50	Manzar et al. (2022)
*Myrtaceae* hosts	*Austropuccinia psidii*	Myrtle rust	70	Du Plessis et al. (2019)
*Oryza sativa* (rice)	*Bipolaris oryzae*	Brown spot of rice	50 to 90	Sobanbabu et al. (2018)
*Buxus* sp.	*Calonectria pseudonaviculata*	Boxwood blight	*100*	Gauthier and Dockery (2018)
Multiple genera of plants	*Colletotrichum* spp.	Anthracnose	50	Manzar et al. (2022)
*Triticum aestivum* (wheat)	*Bipolaris sorokiniana*	Leaf spot	30–40	Kashyap et al. (2023)
*Hevea brasiliensis* (Pará rubber)	*Colletotrichum* spp.	Colletotrichum leaf disease*/*CLD	*80*	Cao et al. (2019)
*Cornus* spp.	*Discula destructive*	Dogwood anthracnose	90	Redlin (1991)
*O. sativa* (rice)	*Entyloma oryzae*	Rice leaf smut	3–70	Mulder and Holliday (1971)
*Camellia sinensis* (tea)	*Exobasidium vexans*	Blister blight	40	Mabbett (2016)
*Coffea* spp.	*Hemileia vastatrix*	Coffee rust	75	Talhinhas et al. (2017)
*Populus* spp.	*Melampsora medusae*	Poplar leaf rust	30 to 50	Feau et al. (2009)
*Glycine max* (soybean)	*Phakopsora pachyrhizi*	Asian soybean rust	10–80	Rincão et al. (2018)
*Musa* spp. (banana)	*Pseudocercospora fijiensis*	Black sigatoka disease (black leaf streak)	*30–50*	Udayanga et al. (2020)
*Triticum* spp.	*Puccinia triticina*	Wheat leaf rust	20	Terefe et al. (2023)
*O. sativa* (rice)	*Pyricularia oryzae*	Rice blast disease	10 to 30	Klaubauf et al. (2014)
*Olea* spp. (Olive)	*Spilocaea oleagina*	Peacock leaf spot	10–20	González-Lamothe et al. (2002)
*Eucalyptus* spp.	*Teratosphaeria* spp.	Leaf blight	20–25	Crous et al. (2015)
*T.* spp. (wheat)	*Urocystis tritici*	Flag smut	5–20	Savchenko et al. (2017)
*C. dactylon*	*Ustilago cynodontis*	Leaf stripe smut	*95*	Kruse et al. (2018)
*Elymus repens*	*Ustilago serpens*	Leaf stripe smut	23–65	Kruse et al. (2018)
*T. aestivum* (wheat)	*Zymoseptoria tritici*	Leaf spot or speckled leaf blotch	*20*	Allioui et al. (2016)
*G. max* (soybean)	*Phakopsora pachyrhizi*	Asian soybean rust	90	Rincão et al. (2018)
Multiple genera of plants.	*Podosphaera xanthii*	Powdery mildew	>50	Zhang et al. (2020)
Soil borne pathogens				
	*Rhizoctonia solani*	Root rot	76	Ghoneem et al. (2023)
	*Fusarium udum*	Fusarium wilt	30 to 100	Ravikumara et al. (2022)
	*Phytopthora drechsleri*	Phytopthora blight	98	Sharma et al. (2023)
	*Macrophomina phaseolina*	Root rot	10–100	Kaur et al. (2013)
	*Fusarium oxysporum* f. sp. *cicero*	Fusarium wilt	50–100	Olszak-Przybyś et al. (2023)
*Cicer arietinum*	*Sclerotium rolfsii*	Collar rot	10–30	Tarafdar et al. (2018)
*Solanum lycopersicum*	*Fusarium oxysporum* f. sp. *Lycopersici*	Fusarium Wilt of Tomato	80–90	Leppla et al. (2004)
*Brassica juncea L*	*Xanthomonas campestris pv. Campestris*	Black rot	10–50	Kesharwani et al. (2022)
*Citrus Jambhiri*	*Xanthomonas citri* pv. *citri*	Citrus Canker	10–50	Mahawer et al. (2023)
*Viral diseases*				
*Nicotiana tabacum*	*Tobacco mosaic virus* (TMV)	Tobacco mosaic	20	Scholthof, (2004)
	*Tomato spotted wilt virus* (TSWV)	TSW disease	60–100	Fletcher et al. (2016)
*Solanum lycopersicum*	*Tomato yellow leaf curl virus* (TYLCV)	TYLC disease	100	Czosnek (2007)
*Cucumis sativus*	*Cucumber mosaic Virus* (CMV)	Cucumber mosaic	80	Palukaitis and García-Arenal (2003)
*Solanum tuberosum*	*Potato Virus Y* (PVY)	PVY disease	10–80	Chung et al. (2008)
*Brassica oleracea*	*Cauliflower mosaic virus* (CaMV)	CaMV disease	25–59	Park et al. (2001)

## Plant disease management benefits and risks

Plant disease control is critical for avoiding production losses in diverse crops. The majority of management techniques fall into three categories: physical, chemical, and biological. Plant disease control relies heavily on the indiscriminate use of chemical pesticides such as fungicides, bactericides, and insecticides that are detrimental to plant pathogens or vectors (Singh et al., 2020). However, the adverse effects of these pesticides and their breakdown products may represent a risk to the environment and human health prompting researchers and producers to investigate alternative and eco-friendly methods of disease management (Ali et al., 2018; 2023). Also, heavy reliance on chemical pesticides may foster the development of pesticide-resistant pathogens. Furthermore, frequent use can disrupt natural ecosystems and beneficial organisms (Pathak et al., 2022). There is a growing emphasis in research emphasize on the development of safer and more efficient fungicides and pesticides, including novel formulations and application techniques. On the other hand, biological control methods harness beneficial organisms to suppress plant pathogens. Using natural enemies, such as predators, parasitoids, and microorganisms, suppresses plant pathogens. It is eco-friendly and sustainable, minimizing the use of chemical pesticides and reducing the risk of pesticide resistance. However, it takes time for populations of natural enemies to establish and control the pathogen, which can pose unintended ecological impacts on non-target species (Kohl et al., 2019). Another important disease controlling tactics in agriculture is implementing cultural practices mitigating plant disease incidence and severity. Practices such as crop rotation, sanitation, and proper irrigation management contribute to disrupting pathogen populations and interrupting their disease cycles. However, cultural practices require significant labour or changes in farming practices, which could increase production costs. Additionally, cultural control alone may not be sufficient to manage severe pathogen infections (Singh et al., 2012). Developing crop varieties with genetic resistance to specific pathogens can offer long-term and sustainable protection. This approach reduces the dependence on chemical pesticides and keeps pathogens at bay (Pathania et al., 2021). However, developing resistant varieties through breeding or genetically engineered can be time-consuming. Additionally, pathogens may evolve to overcome plant resistance, rendering resistance ineffective over time (Miedaner, 2016). However, genome editing (GE) methods enable quick trait alteration in agricultural plants and has emerged promising tool for developing disease resistant crops.

## Methods for developing disease-resistant plants

One of the primary goals of plant pathologists is the identification and exploitation of potential targets that have evolved in disease resistance mechanisms that can be utilized for generating future disease resistance cultivars in sustainable agriculture. This requires an in-depth understanding of the molecular mechanisms of host-pathogen interactions and disease resistance. Plants evolved multi-layered defensive systems against microbial infections over time. Pre-formed physiological barriers play the first line of defense against microbial pathogens; however, plants may also generate adequate defensive responses after pathogen perception, which is mediated by cell wall plasma membrane-bound and intracellular immunological receptors that identify pathogen-derived molecules or by indirectly modifying host targets. With the advent of high-throughput tools, the identification and functional validation of genes related to host immunity, such as receptors, pathogen Avr proteins and effectors, pathogen-associated molecular patterns (PAMPs), channels, and signaling molecules, has provided a snapshot of plant immune systems and their signaling cascades in response to different pathogens. Now it is well documented that there are two tiers of plant immune systems: effector-triggered immunity (ETI) and PAMP-triggered immunity (PTI) (Jacob et al., 2013). Modifying or engineering genetic resistance traits is the most cost-effective strategy to improve disease resistance in crops. However, this requires vast knowledge about the molecular dynamics of host-pathogen interactions and plant immune responses. For developing long-term disease-resistant cultivars, there is a need to understand host-pathogen interaction using different tactics. For example, an in-depth understanding of disease epidemics and “hot spots” to screen disease-resistant or susceptible genotypes in the field during natural disease epidemics is important in order to choose resistant genotypes. After being cultivated in hotspot areas, the test genotypes can be examined for their disease resistance or susceptibility (Ye et al., 2002). Major obstacles that are often faced in the natural screening method could be avoided by screening disease-resistant genotypes under artificial circumstances (Ye et al., 2002). Genotypes can be screened in a glasshouse with controlled environmental conditions to monitor disease resistance using new tools. In the controlled glasshouse or greenhouse, the ideal photoperiod, relative humidity, and temperature may be readily changed to meet the needs for the advancement of the disease. Certain laboratory-based screening techniques, such as the whole plant infection, detached leaf test, purified phytotoxins or culture filtrates, and the cut-twig method, are commonly used to study host pathogen interactions and disease progression (Sharma et al., 1995).

In the past, traditional breeding and transgenic breeding have played key roles in improving disease resistance in various economically important crops. Like selecting resistant plants from commercial varieties, the utility of this method is limited. Introduction of resistant varieties into new areas; however, this method possesses the limitation of variety not performing well in new environments or becoming susceptible to specific diseases in new areas (Russell, 2013). By mating a superior recipient parent line with a donor line that possesses advantageous qualities, conventional plant breeding, predominantly cross-breeding, has long been used to enhance plant properties, including disease resistance. However, this whole method is labor-intensive, time-consuming, ineffective in transferring undesired genes to desired genes and rife with drawbacks (Gao, 2021). Another mainstay of disease resistance breeding programs is the establishment of appropriate and efficient screening methods. To achieve desired results, a thorough understanding of pathogenicity, virulence pattern, resistance type, and an efficient breeding strategy is needed. Effective strategies for screening disease resistance to multiple pathogens have been developed, and enough research has been conducted in the past 10 years to define the nature and durability of resistance (Podder et al., 2013). The development of molecular markers and next-generation sequencing techniques made it simple to pinpoint the genes and QTLs linked to particular disease resistance. Recent genomic techniques, such as bi-parental QTL mapping (Collard et al., 2005), association mapping and QTL Seq (Takagi et al., 2013), can be used to correctly quantify the connection between genes and molecular markers. Creating a mapping population, screening for polymorphic markers, phenotyping the population, genotyping the population using polymorphic markers, creating a linkage map, performing QTL analysis, and validating linked markers are all steps in the development of molecular markers. To screen a candidate, a molecular marker closely associated with the QTL or gene of interest may be used.

Under incompatible host-pathogen interactions, the host displays complete disease resistance against the pathogen when both the pathogen’s homologous avr and the host’s R gene are present (Flor, 1971). In the early 1900s, British scientist Rowland Biffen showed that R gene-mediated resistance might be beneficial in wheat (*Triticum* sp.) breeding (Biffen, 1905). Since then, a large number of R genes have been cloned and inserted into other species (De Wit et al., 1985), genera (Tai et al., 1999), and species borders (Song et al., 1995). For instance, in laboratory settings, the transfer of the R gene *Rxo1* from maize (*Zea mays*) into rice (*Oryza sativa*) resulted in resistance to the bacterial streak disease (Zhao et al., 2005). Multiyear field trials conducted in tomatoes in commercial-type growth settings have shown that strong resistance to *Xanthomonas* sp. producing bacterial spot disease is conferred by tomatoes expressing the pepper gene (Kunwar et al., 2018). In the field, wheat transgenic for different alleles of the wheat resistance locus Pm3 showed racialized resistance to powdery mildew (Brunner et al., 2012). Strong field resistance to the causative agent of potato late blight, is exhibited by transgenic potatoes expressing the wild potato R gene RB or Rpi-vnt1.1 (Jones et al., 2014). Notably, the only genetically modified crop with increased resistance to a nonviral disease that has been licensed for commercial use to date is the transgenic potato expressing created by J.R. Simplot.

As pathogens can escape detection of their gene by the *R* gene, the disease resistance conferred by a single *R* gene is therefore frequently not durable in the field. Multiple *R* genes are sometimes inserted simultaneously, a practice known as stacking, to increase durability and widen the resistance spectrum (Mundt, 2018). Since the emergence of a pathogen strain that may overcome resistance supplied by several genes at once is a rare occurrence, resistance conferred by stacked *R* genes is projected to be long-lasting. Cross-breeding pre-existing R loci is one method of stacking *R* genes. Breeders can use marker-assisted selection to identify offspring with the desired R gene makeup (Das and Rao, 2015). For instance, three *R* genes—*Xa21, Xa5, and Xa13* give resistance to *X. oryzae* pv. *oryzae* (Xoo) in the deep-water rice cultivar Jalmagna rice by cross-breeding and marker-assisted selection (Pradhan et al., 2015). Eight Xoo isolates were investigated, and the line that resulted with the stacked *R* genes displayed a high degree of disease resistance under field conditions (Pradhan et al., 2015). Even though marker-assisted selection has significantly improved selection efficiency, integrating several loci using this technique may still take a long time. Only a few quantitative traits controlling a remarkable variation in population can be selected by this method (Lamichhane and Thapa, 2022).

In chemical or physical mutagenesis, seeds are exposed to various mutagens to cause mutations in the plant’s DNA. This process is known as mutation breeding. The process’s mutants go through rigorous selection to assess desired traits and find new genes in the genome. However, mutant breeding is a laborious and drawn-out procedure that frequently takes 6–7 years to achieve the desired outcomes. The randomness of mutations, which makes their detection and prediction difficult, is a key methodology drawback. Several crops have effectively enhanced a variety of qualities through transgenic breeding, which requires moving a desired gene from one genome to another; examples include Bt cotton, Bt maize, transgenic rice resistant to blast, sheath blight, and false smut (Shen et al., 2023). To improve desired features, the gene of interest is carefully introduced into the host variety’s genome. However, the introduction of foreign DNA causes transgenic crops to be genetically changed, which raises concerns among the general public and the scientific community about their adoption (Ahmad et al., 2020).

Genome editing is one of the new genetic modification approaches that is sometimes attributed to “new breeding techniques,” and it offers encouraging availabilities for the sustainable development of crops in the future. With the use of gRNA and guided target sites, genome editing enables the exact targeting and destruction of certain negative regulators or genes, as well as the reorganization of chromosomes in elite variety genomes. The utilization of genome editing approaches, such as the CRISPR/Cas9 system, offers benefits including efficacy, robustness, cost-effectiveness, and the lack of foreign DNA, potentially enabling these crops to circumvent GMO restrictions and be marketed as non-GMOs (Ahmad et al., 2020).

## Microbial pathogen evades plant immunity

Plant pathogens demonstrate diverse strategies, ranging from biotrophic to necrotrophic lifestyles, to efficiently colonize plant hosts (Andolfo and Ercolano, 2015). Biotrophs rely on live host cells, either completely or partially, for their life cycle completion, causing minimal damage to host cell walls and maintaining host viability. Examples include obligate biotrophic fungi like *Puccinia graminis*, which depend on living host cells for sustenance. In contrast, necrotrophs such as *Botrytis cinerea* kill host tissues during infection, using cell wall-degrading enzymes and toxins to obtain nutrients. These pathogens deploy various virulence factors, including effectors, toxins, and cell wall-degrading enzymes, to manipulate host physiology and evade immune recognition. Nevertheless, plants have developed sophisticated defense mechanisms to counter pathogen attacks. The plant immune system utilizes pattern recognition receptors (PRRs) to detect conserved microbial signatures, initiating immune responses mediated by defense hormones like salicylic acid, jasmonic acid, and ethylene. Additionally, plants harness resistance (R) genes, encoding intracellular receptors that specifically recognize pathogen effectors, leading to a localized hypersensitive response (HR) and systemic acquired resistance (SAR). Understanding this intricate interplay between pathogens and plant immunity is pivotal for comprehending disease dynamics and devising crop protection strategies (Jones and Dangl, 2006; Andolfo and Ercolano, 2015).

Plants lack an adaptive immune system, unlike animals (Kumar et al., 2011). Consequently, they rely on a complex array of defences to fend off pathogens. However, pathogens have evolved strategies to bypass or suppress these plant immunity mechanisms. Here’s how they accomplish this: The primary defense line for plants is the cell wall, a robust barrier primarily composed of cellulose and other constituents. Pathogens employ various tactics to subdue this first line of defense, known as pattern-triggered immunity (PTI). Some produce enzymes that degrade these components, facilitating entry. For example, fungal pathogens release enzymes like cutinases, cellulases, and pectinases to breach the plant cell wall. An instance is seen in Colletotrichum gloeosporioides, the pathogen responsible for oil tea anthracnose, which secretes the cutinase CglCUT1 to degrade the cuticle during infection (Wang et al., 2017). Others release molecules that mimic plant hormones, manipulating plant growth and impeding defense responses. *Pseudomonas syringae*, for instance, produces effectors that manipulate stomata, aiding pathogen entry (Zhang et al., 2022). Pathogen effectors can also target plasmodesmata (PD), enlarging pore size to control cytoplasmic continuity. During Fusarium oxysporum infection, effectors like Avr2 and Six5 enlarge PD pore size (Cao et al., 2018). *Xanthomonas oleifera* T3E XopR hijacks the Arabidopsis actin cytoskeleton during infection, disrupting host actin assembly (Sun et al., 2021). In a study by Jin et al. (2023), the bacterial pathogen *P. syringae*’s T3SS protein HrpP was found to facilitate effector translocation and manipulate plant immunity to promote bacterial infection. This underscores the ongoing arms race between plants and pathogens, where both constantly evolve strategies to outmaneuver each other. Pathogens often target receptor-like kinases to avoid recognition by the plant’s immune system. Effector proteins like CoNIS1 and MoNIS1 inhibit kinase activity, thereby suppressing immune activation (Irieda et al., 2019). Pathogens also act through effectors to disrupt phytohormone-dependent plant defenses. For instance, oomycete pathogens secrete isochorismatases that hinder salicylic acid (SA) biosynthesis, diminishing the plant’s ability to mount an effective defense response (Han et al., 2019). By employing these tactics, pathogens establish themselves within the plant and cause disease. Understanding these mechanisms is vital for developing strategies to protect crops and enhance plant disease resistance.

## Genome editing (GE) technology for improving plant traits

**FIGURE 1:**
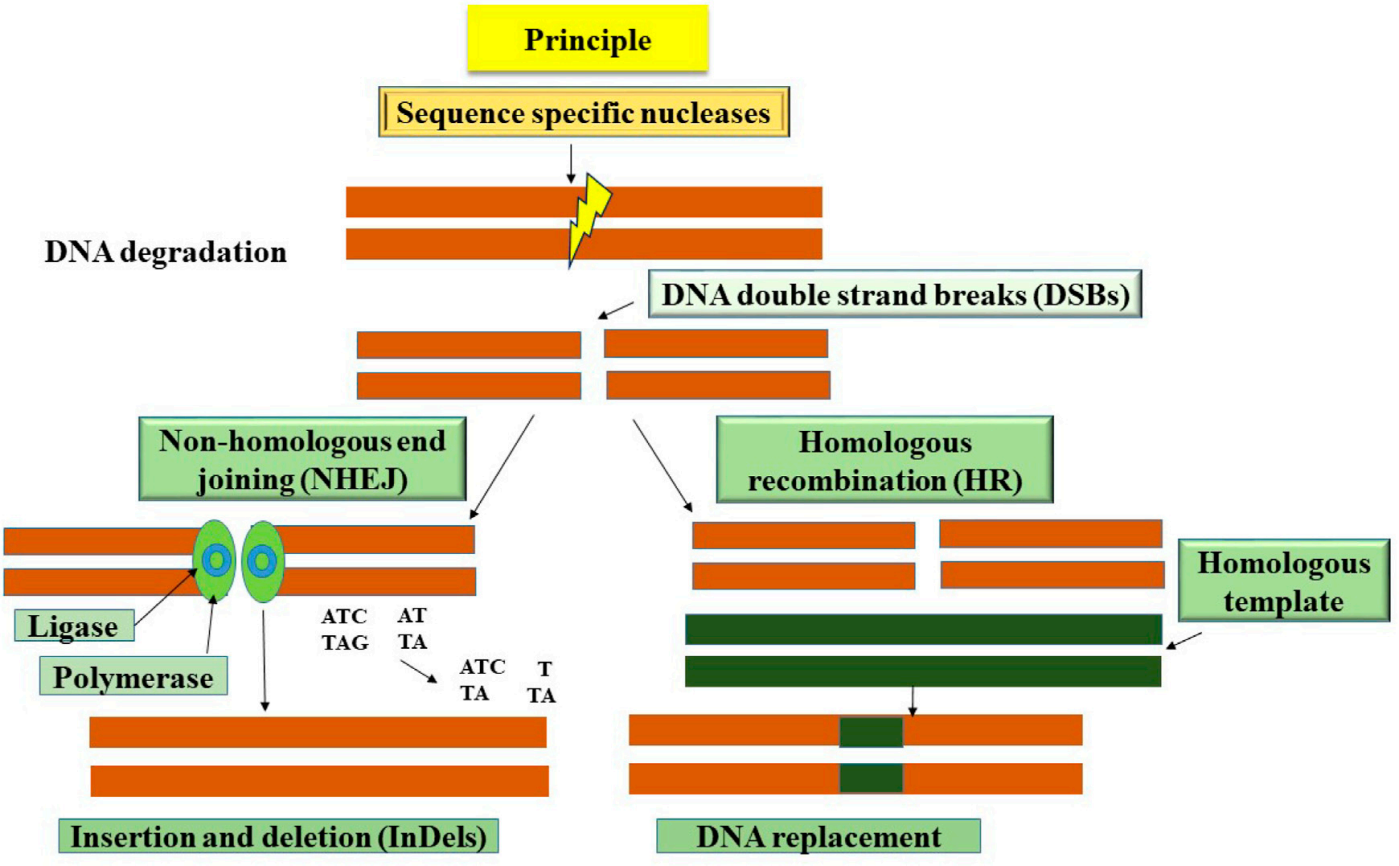
A schematic representation shows the basic principle of genome editing techniques to cleave double-stranded DNA at a targeted position on the genome. Various methods such as insertions, deletions or substitutions or gene insertion are used to disrupt the function of the gene using genome editing.

Genome editing refers to the exact alterations made to the genomic DNA of cells or animals using site-specific genome targeting methods, similar to targeted mutagenesis or site-directed insertion, deletion, or replacement (Ahmad et al., 2020). These cutting-edge methods have a great deal of promise to be effective tools for understanding gene function and promoting crop development. Sequence-specific nucleases are used in the procedure to recognize and target certain DNA sequences, resulting in the creation of double-stranded breaks (DSBs) at predetermined places. Diverse mechanisms, including non-homologous end-joining (NHEJ), micro-homology-mediated end-joining (MMEJ), and homology-directed repair (HDR) pathways, are used to restore DSBs (Figure 1) (Molla and Yang, 2020). Although NHEJ is the main DSB repair pathway, it frequently introduces mistakes that lead to insertion or deletion changes at the DSB sites. Conversely, while it does not happen often in plant systems, repair via the HDR route is error-free. CRISPR/Cas9 and other gene editing technologies, in particular, have a dramatic impact on fundamental research in plants and crop enhancement. Notably, one of the key benefits of these methods is the simplicity with which the transgenes originally used for genetic alteration may be removed by genetic segregation. As a result, gene-edited variants become identical to those created utilizing traditional breeding techniques (Zhang et al., 2017). By focusing on illness-related genes, genome editing has been used to ameliorate disease resistance (Zhang et al., 2017). Additionally, genome editing has been used to build broad-spectrum disease resistance in crops. This has been done either by using particular host-vulnerability genes (the S gene method) or by cleaving the genetic material of phytopathogens to prevent their growth (Zaidi et al., 2018).

## Applications of genome editing technologies

Single gene knockout and multiplex gene knockout stand out as two particularly influential uses of genome editing in modern plant science. Individual gene knockout: currently, precise deletion of target genes is the remarkably common and generally used use of genome editing. This method enables thorough investigations into gene activities and their consequences in diverse biological processes by selectively disrupting particular genes. Multiple genes may be targeted concurrently in different plant species via genome editing, a technique known as multiplex gene knockout. Researchers may explore complicated gene connections and regulatory networks using this multiplex gene knockout technology, providing greater insights into the intricacies of plant biology (Mushtaq et al., 2019). Large-scale deletions: When two double-stranded breaks (DSBs) are established on the same chromosome at a specific distance, the two points can be connected via the non-homologous end-joining (NHEJ) repair pathway, which results in the deletion of the intervening sequence. For particular research projects and crop development initiatives, similarly to the investigation of gene clusters and noncoding RNAs, this method of generating rather significant deletions has proven useful (Ordon et al., 2017). Gene knockout in polyploid plants: In polyploid crops lacking sufficient mutant resources, genome editing methods have shown outstanding value. Notably, genome editing tools have accomplished gene knockout in triploid plants, with citrus and apples serving as examples (Zhang et al., 2017). This has permitted precise genetic alterations in complicated polyploid genomes. Gene targeting is the deliberate use of genetic engineering techniques to substitute a DNA fragment exactly for another (gene replacement) or to precisely introduce a new sequence into a particular genomic location (gene knock-in) (Zhang et al., 2017). This focused strategy has enormous potential for specialized genetic alterations and might revolutionize crop improvement and other genetic treatments. In the past, the application of genome editing has been carried out in both model species *Arabidopsis thaliana* (Li et al., 2013) and *Nicotiana benthamiana* (Gao et al., 2015), as well as different crop systems such as rice, wheat, sorghum, soybean, corn, and potato, to improve diverse traits related to stress tolerance, growth, and yield (Rani et al., 2016). Similarly, by altering disease-related genes, genome editing has shown promise for improving disease resistance and providing a way to strengthen crop defences against pathogens (Mishra et al., 2021).

## Genome editing for disease resistance: the choice of gene or genomic loci

**FIGURE 2:**
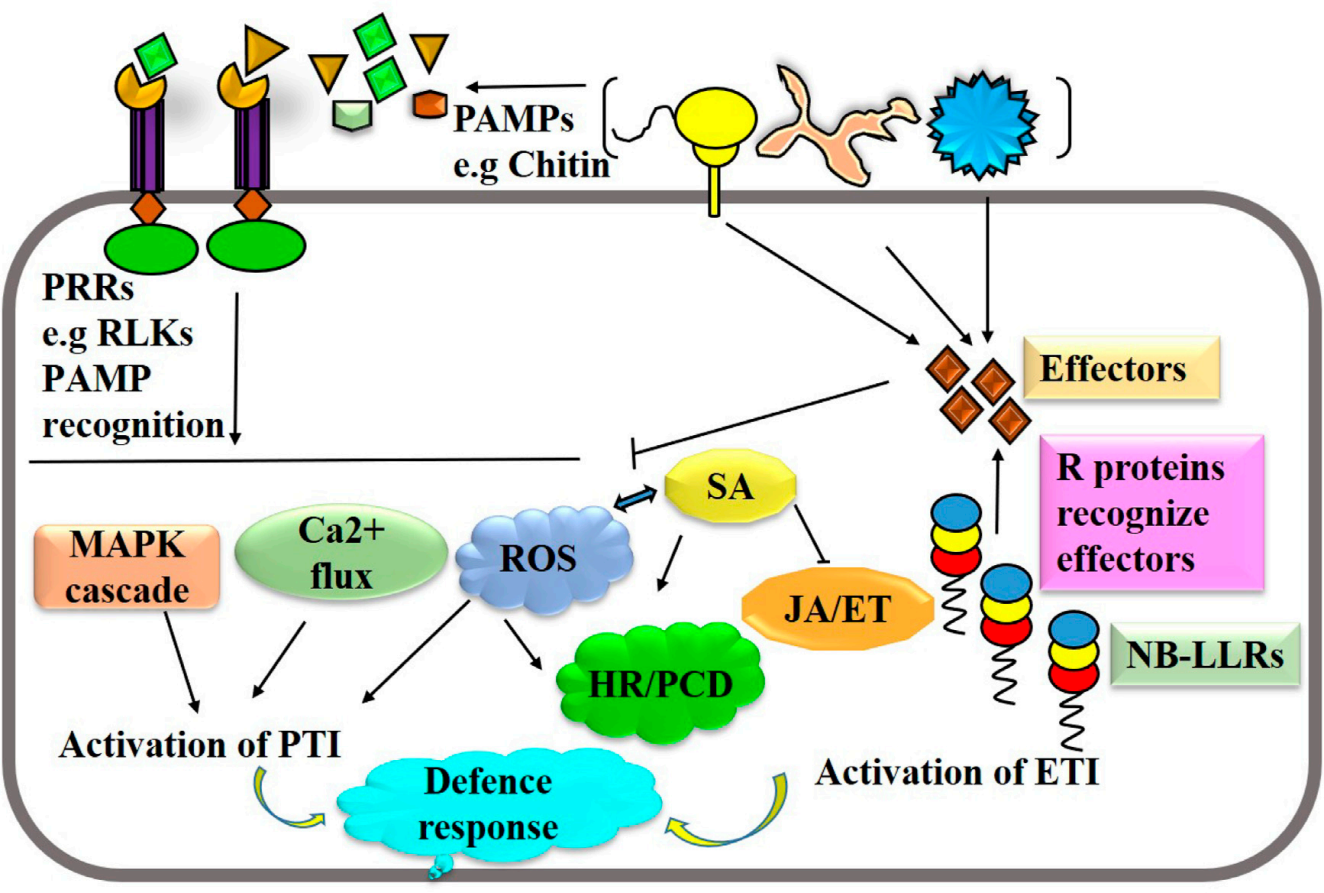
A simplified schematic illustration of the immune system in plants. Recognition of pathogen or derived elicitors by pattern-recognition receptors and effectors by nucleotide-binding leucine-rich repeat leads the activation of PTI and ETI. Activation of PTI and ETI lead a series biochemical events like cytosolic calcium burst, ROS formation and hormonal reprogramming that regulates plant immune responses.

Plants defend against pathogen attacks by two-tiered immune systems, namely, PTI and ETI, that are triggered by cell surface-localized PRRs and intracellular nucleotide-binding domain leucine-rich repeat-containing receptors (NLRs) (Ali et al., 2018). Both PTI and ETI immune receptor classes eventually converge into many identical downstream responses, even though with differing amplitudes and dynamics; this is despite the fact that they involve separate activation processes and appear to require different early signaling components. Different tactics are employed by plants to fend against pathogen attacks. Immune receptors such as extracellular pattern-recognition receptors (PRRs) and nucleotide-binding leucine-rich repeat (NLR) receptors recognize pathogens or their derived molecules or effectors, which triggers a variety of defense signaling pathways to thwart pathogen attack (Figure 2). Activation of PTI and ETI lead a series biochemical events like cytosolic calcium burst, ROS formation and hormonal reprogramming that regulates plant immune responses. Genome editing can be used to manipulate several potential targets, of host immune system and pathogens including effector binding targets, S and R genes, hormonal pathways, and pathogen virulence factors, in order to improve disease resistance. Phytopathogens usually target susceptibility (S) genes in plants in order to multiply and cause disease progression more easily. Therefore, by altering these S genes, it may be possible to create long-lasting, broad-spectrum disease resistance by interfering with the host-pathogen relationship. Targeting S genes using genome editing is the most viable and transgenic-free approach to developing disease-resistant cultivars in sustainable agriculture. For instance, editing susceptibility genes like SWEETs (Sugars Will Eventually Be Exported Transporters) or receptor-like kinases (RLKs) can reduce pathogen susceptibility (Gupta et al., 2021). Additionally, manipulating defense-related genes like transcription factors or genes involved in hormone signaling pathways can bolster plant immunity (Kourelis and Van der Hoorn, 2018). On the pathogen side, editing virulence factors such as effectors or genes involved in pathogen recognition can attenuate pathogenicity. For example, CRISPR-mediated editing of effector genes in pathogens like Phytophthora infestans can impair their ability to cause disease (Fang and Tyler, 2016). Furthermore, targeting essential genes in pathogens through gene editing can render them non-viable or less virulent, thereby reducing disease severity (Nekrasov et al., 2017). Integrating these approaches offers a promising avenue for developing durable disease resistance in crops.

## Types of genome editing techniques

Modern genome editing (GE) technologies have been instrumental in crop improvement, especially in terms of boosting disease resistance. The CRISPR/Cas9 system, CRISPR/Cas13, base editing, transcription activator-like effector nucleases (TALENs), zinc-finger nucleases (ZFNs), and meganucleases are some of these cutting-edge techniques which have been utilized from crop improvement (Karmakar et al., 2022). With the use of these versatile tools, crop genomes may be precisely and specifically modified, opening up the enormous potential to engineer disease resistance in crops by changing certain genes involved in the plant immune system. They are useful tools to improve crop food sustainability and security throughout the world because of their efficacy and adaptability. In this review, we discussed the application and limitations of different genome editing tools available for crop improvement.

## Meganucleases and ZFNs (zinc finger nucleases)

One of the earliest sequence-specific nucleases used for targeted double-stranded DNA breaks was the meganuclease, also known as the homing endonuclease. Prokaryotes, archaea, and unicellular eukaryotes all include them naturally (Silva et al., 2011). These amazing enzymes are highly effective at cleaving double-stranded DNA at predetermined recognition sites, which generally include between 14 and 40 base pairs. Their recognition sites are larger than those of type II restriction enzymes frequently used in recombinant DNA technology, making them the most popular naturally occurring restriction enzymes (Carroll et al., 2017). Meganucleases may change their recognition sequence through protein engineering, allowing them to replace, remove, or change any sequence of interest in an extremely focused and effective manner. They are involved in the process of homing, which permits them to start recombination, and are encoded by mobile genetic elements. These genes can transmit genes horizontally because they are located in introns or intergenic regions (Stoddard, 2005). Meganucleases, which include DNA binding and DNA cleavage domains, cause double-stranded breaks at their recognition sites. Homologous recombination (HR) is then used to repair the lesions. By inserting the meganuclease coding sequence into the target gene, targeted genome engineering with designed meganucleases is made possible (Hafez and Hausner, 2012). Meganucleases have been used to precisely alter plant genomes since the 1990s and to learn more about the underlying principles controlling the integrity of plant genomes. However, their application in genome engineering is not as general as that of zinc-finger nucleases (ZFNs) or transcription activator-like effector nucleases (TALENs) due to certain limitations. Firstly, the DNA binding and cleavage domains in meganucleases overlap, which can compromise their catalytic activity (Stoddard, 2011). Secondly, meganucleases lack the modular DNA-binding domain architecture present in ZFNs and TALENs. Lastly, there are instances where meganucleases may exhibit sequence degeneracy, increasing the likelihood of off-target binding (Argast et al., 1998). Despite these limitations, meganucleases remain a promising avenue for targeted genome engineering and persist as a subject of research and development in the field of genetic modification.

The first sequence-specific nucleases previously used for genome editing in eukaryotes were zinc finger nucleases (ZFNs). ZFNs were used for the first time to modify the plant genome in 2005 (Lloyd et al., 2005). One of the most frequent DNA-binding domains in eukaryotes is the zinc finger domain, which was first determined in 1985 as a component of transcription factor IIIa in *Xenopus* oocytes. The zinc finger (ZF) domain is made up of a variety of Cys2His2 zinc fingers, each of which has around 30 amino acids and may bind to homologous triplets of nucleotides. The ability of ZFs to recognize all 64 potential nucleotide combinations makes it possible to develop ZFNs that can specifically target any 3–6 nucleotide triplet. There are two distinct functional groups in the ZFN monomer. A synthetic ZF domain at the N-terminus attaches to the target DNA, and a DNA cleavage domain called FokI is located at the C-terminus, where it causes double-strand breaks (DSBs) in the target genomic region. The FokI nuclease must dimerize to break double-stranded DNA. To create a DSB at the targeted DNA site, a set of ZFNs is necessary. Two distinct ZFNs must bind to the forward and reverse strands independently for FokI dimerization to occur (Zhang et al., 2017). Additionally, the forward and reverse target sequences should be separated by a 5–7 bp spacer region. ZFNs have been commonly employed for genome editing in both plant and animal systems for more than 10 years (Mushtaq et al., 2019). ZFNs have been used to change the endogenous gene ABA-INSENSITIVE-4 (ABI4) in Arabidopsis, for example, (Osakabe et al., 2010). ZFNs were employed in the deletion of a 4.3 kb integrated GUS gene sequence in tobacco that was surrounded by ZFN cleavage sites (Petolino et al., 2010). Additionally, ZFNs were used to target three homologous copies of the aceto-hydroxy acid synthase (AHAS) gene in hexaploid wheat (*T.* aestivum) to simultaneously knock down several genes (Ran et al., 2018). ZFNs have been successful in genome editing, but their design and aggregation are technically difficult, and it can be expensive to outsource the production of ZFN modules to commercial vendors (Ramirez et al., 2008). Despite these obstacles, ZFNs have significantly contributed to the evolution of genome editing and have been used in several ground-breaking investigations involving a variety of taxa.

## TALENs are transcription activator-like effector nucleases

When compared to ZFNs, TALENs, which are protein-based DNA-targeting enzymes, have been shown to be more efficient and selective. Like ZFNs, TALENs are made up of two domains: a target-specific DNA-binding domain that may be customized and a non-specific DNA cleavage domain (Fok1). The TAL effector (TALE) is the name of the DNA-binding domain of TALENs. When *Xanthomonas* bacteria infect their host plants, they release TAL effector proteins that bind to the plant’s DNA via a domain that contains some tandem 34–35 amino acid repeats (Malzahn et al., 2017). An N-terminal translocation signal, an acidic transcription-activation domain, and a central DNA-binding domain are all common features of TALE proteins. The repeat variable di-residues (RVDs), which are located next to each other at positions 12 and 13, make up the almost identical 33–35 amino acid repeats that make up the DNA-binding domain. Two different teams independently cracked the DNA binding specificity of TALE in 2009 (Moscou and Bogdanove, 2009). The DNA-binding domain’s repeat units each bind to a single nucleotide, and the RVDs inside the repeats have a significant connection with one particular nucleotide (A, G, C, or T). By choosing a certain aggregation of the four conspicuous repetitions to accommodate the proper RVDs, it is possible to create particular DNA-binding proteins thanks to the clear link between protein sequence and DNA recognition. Since TALENs are methylation-sensitive, they may be created and come together to target any DNA sequence (Kaya et al., 2017b). Similar to ZFNs, a dimerized functional FokI requires two Talen monomers to form. Since TALENs are simpler to assemble than ZFNs, more plants are using this editing technique (Zhang et al., 2017). Designing a sizable number of identical repeat sequences for TALENs, however, is one of the main technological hurdles addressed by scientists (Li et al., 2020). By accurately altering the Lipoxygenase (Lox3) gene, TALEN-based targeted mutagenesis has been effectively used to improve storage tolerance in rice (Ma et al., 2015).

## CRISPR/Cas9 and CRISPR/Cas13

**FIGURE 3:**
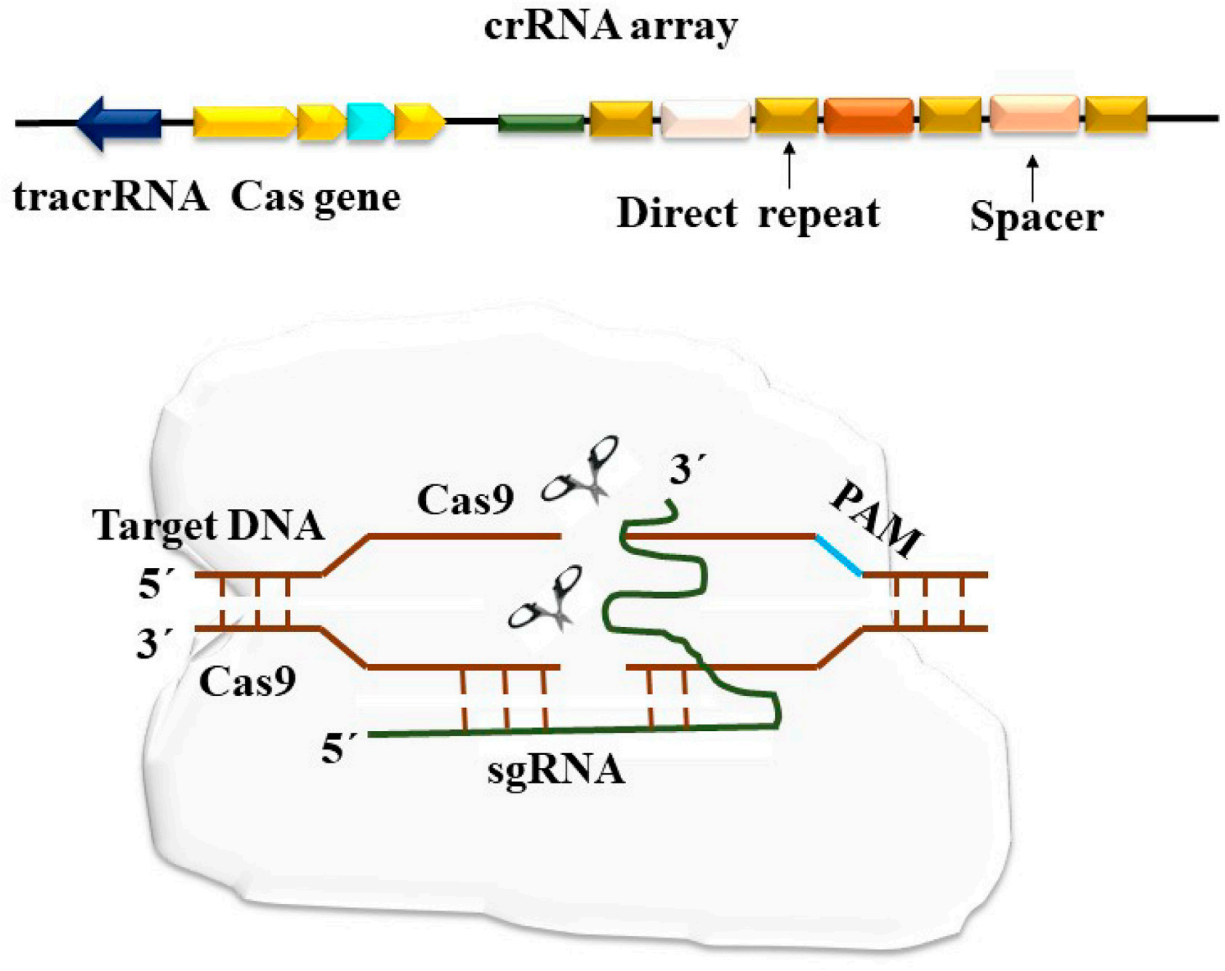
The schematic representation of the CRISPR/Cas9 system depicting a cluster of regularly spaced short palindrome repeats (CRISPR) and the CRISPR-associated nuclease 9 (Cas9). This system operates by inducing double-strand breaks (DSB) in the DNA strand. The Cas9 protein is directed by CRISPR RNA (crRNA) to perform its molecular function.

Early 1987 witnessed the discovery of CRISPR, which has subsequently been found in numerous more bacterial species. However, CRISPR was first used to alter plant genes in 2013, and it has since taken over as the most extensively used gene editing approach (Nekrasov et al., 2017). Depending on how the Cas genes are arranged and their structural variation, the CRISPR-Cas system may be divided into two main groups. While Class 2 CRISPR-Cas systems are characterized by a single effector complex, Class 1 CRISPR-Cas systems use several protein effector complexes. Based on recent studies, there are two classes, six kinds, and more than 30 subtypes of CRISPR-Cas systems (Koonin and Makarova, 2022). Although there are many other CRISPR-Cas systems in nature, the Type II CRISPR-Cas9 system from *Streptococcus* pyogenes is the most straightforward. It uses two short RNAs for target identification and a single Cas9 endonuclease to cause double-strand breaks (DSB) in DNA (Liu et al., 2015). Prokaryotes are naturally home to CRISPR- and CRISPR-Cas systems, which act as a defense mechanism against mobile genetic elements like plasmids and phages (Figure 3). There are two primary parts to these systems. The first is a genomic region known as CRISPR, which has a collection of brief foreign-origin sequences known as spacers. These spacers allow the organism to recognize certain mobile genetic elements (MGEs) that it has previously encountered (Vercoe et al., 2013). Repeats, which are repeating regulatory sequences, separate the spacers. A key component of the CRISPR-Cas system is the CRISPR array, which comprises the leader sequence, spacers, and repeats (Yosef et al., 2012). The Cas proteins, which are often present close to the CRISPR array, are the second component of CRISPR-Cas systems. They are encoded by Cas genes. Effectors are components of the CRISPR-Cas system. Three stages make up the mechanism of CRISPR-Cas systems: adaptation, maturity, and interference. New spacers are added to the CRISPR array’s leader end during adaptation. The CRISPR array is translated through the maturation phase, producing pre-CRISPR RNA (pre-crRNA). The pre-crRNA undergoes further processing to become CRISPR RNA (crRNA) molecules, each of which has a single spacer and fragments of a repeat sequence on each side (Burmistrz et al., 2017). RNP complexes are created when these crRNAs join forces with Cas proteins. According to Semenova et al. (2011), the RNP complexes search nucleic acids for a sequence that matches the one encoded by the crRNA. The interference phase begins when the target sequence is recognized, which causes the recognized nucleic acid to degrade. In almost half of the known bacterial and archaeal species, CRISPR-Cas systems are present in the prokaryotic world (Van der Oost et al., 2014). Depending on the design of the effector complex, they are now divided into two groups (Makarova et al., 2018). While class 2 systems use single-subunit RNP complexes, class 1 RNPs have many subunits. Based on particular Cas proteins and the design of the CRISPR loci, each class is further classified into kinds and subtypes. Modern molecular biology has been changed by the discovery of CRISPR-Cas systems, which provide several desirable characteristics for future tools in this field. Due to the modular design of the CRISPR locus, it displays excellent specificity when recognizing target sequences, which can be readily changed by changing the crRNA sequence. The breadth of uses for CRISPR-Cas systems is also increased by altering the Cas proteins themselves (Brezgin et al., 2019). The CRISPR-Cas9 system stands out as the most practical foundation for the enlargement of new tools in research and biotechnology, even though the majority of the documented CRISPR-Cas systems target DNA sequences (Khosravi, 2021). However, the recent identification of RNA-targeting CRISPR-Cas systems offers fresh opportunities for the creation of novel research and biotechnology instruments.

**FIGURE 4:**
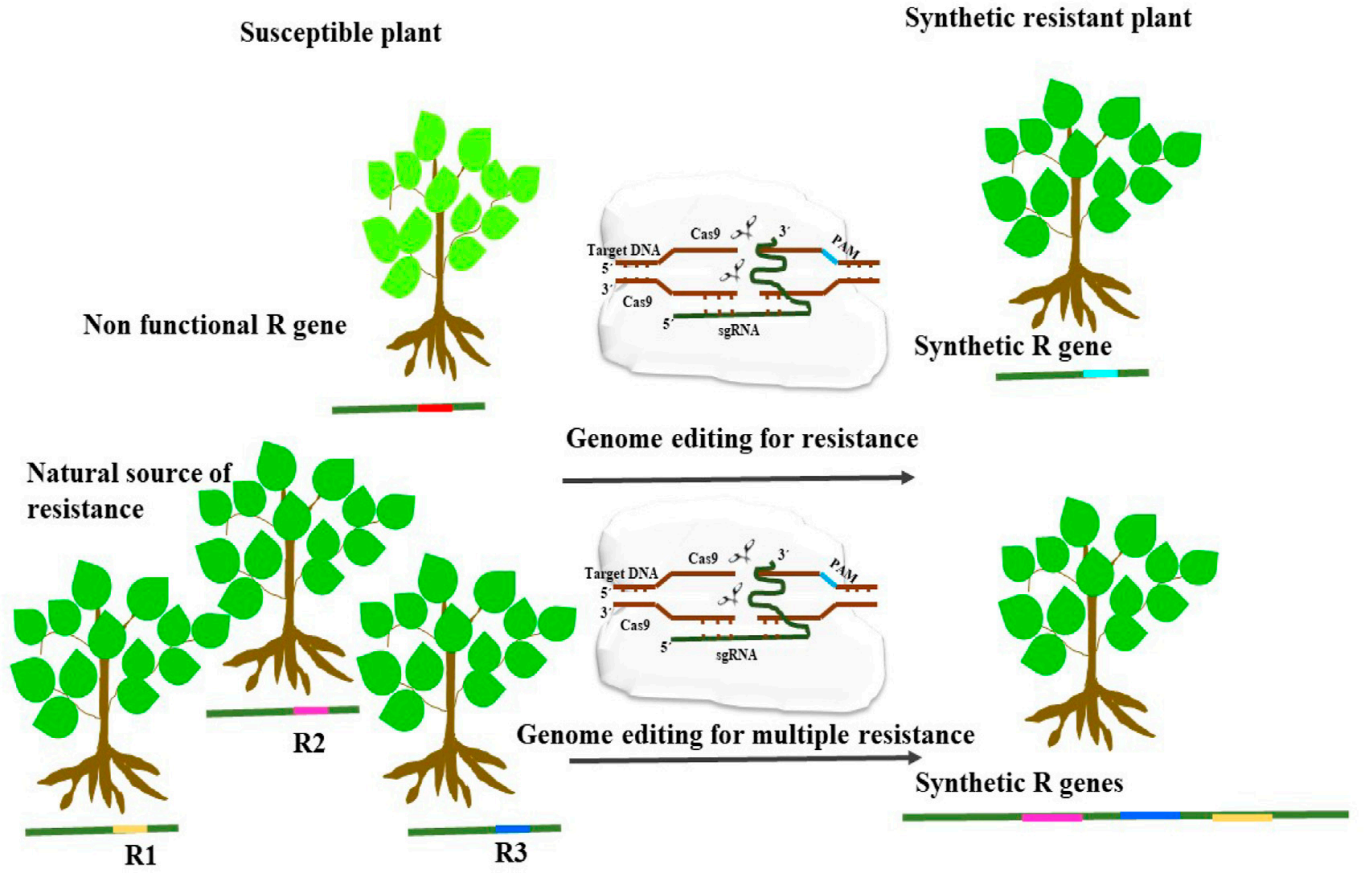
An illustration showing the application of genome editing for enhancing disease resistance by modifying non-functional pathogen recognition sites to synthesize synthetic functional R-gene. Another strategy is to engineer R gene for detecting multiple pathogen recognition sites which could provide resistance to several pathogens.

The following procedures are frequently used to modify the plant’s genome: First, guide RNAs (sgRNAs) are generated manually or with the use of online tools. They have a 20-bp target sequence at the 5′ end. Second, the kind of PAM sequence depicted in the target genomic area is used to determine which Cas variant is most appropriate (Molla et al., 2020). Thirdly, although this step is optional to reduce the possibility of failure, the effectiveness of the guide RNAs can be evaluated *in vitro* or using a transient protoplast transfection technique. Fourthly, a binary vector made up of one or more sgRNA expression cassettes and a Cas9 expression cassette is created. With this binary vector, biolistic transformation or agrobacterium-mediated transformation can be employed. Fifthly, either the agrobacterium or biolistic method are used to carry out genetic transformation. Sixth, the potential genome-edited plants are regenerated once the changed cells have been suitably chosen. Seventh, to verify effective genome editing in the target region, the putative plants are genotyped using a variety of techniques. The genome-edited plants can be rendered transgene-free by undergoing sexual segregation with the genetically altered plants when effective genome editing has been identified. To assist in the creation of guide RNAs, the choice of suitable editing tools, and the analysis of experimental data, several online applications have been developed (Figure 4). Precision genome and protein engineering is now possible thanks to the considerable advancement of CRISPR-Cas9-mediated gene editing brought about by the growth of bioinformatics-based design tools. Novel applications such as genomic disruption, transcriptional regulation, base editing, epigenetic modification, and prime editing have emerged as a result of this advancement (Molla et al., 2020). Base editing and prime editing technologies are two of the most promising toolkits for precise genome change, having the ability to create desired features in agricultural plants among the sophisticated genome editing methods (Molla et al., 2021). These methods open up a new prospect for precise genetic alterations in plants by overcoming the drawbacks of traditional CRISPR-Cas tools and low-efficiency HDR-mediated genome editing.

Cas13 enzymes have emerged as significant participants in the CRISPR field. Just a year after Cas13a (C2c2) was identified as an RNA-targeting CRISPR enzyme by Feng Zhang’s lab (Abudayyeh et al., 2019), they further advanced the technology by adapting Cas13b for exact RNA editing (Cox et al., 2017). The first RNA editing CRISPR tool was this ground-breaking technology, named REPAIR (RNA editing for programmed A to I (G) replacement). The group then unveiled RESCUE, an RNA editor that permits C-to-U changes, ushering in yet another development in the area. Compared to conventional DNA editing methods, RNA editing has many benefits. Since RNA editing does not need homology-directed repair (HDR) machinery, it may be used in cells that are not dividing. In addition, Cas13 enzymes are more adaptable than Cas9 and Cpf1 enzymes because they do not need a protospacer adjacent motif (PAM) sequence at the target locus. Some Cas13 enzymes favor targets with particular single-base proto-spacer flanking site (PFS) sequences, but LwaCas13a does not. Notably, direct genome editing is impossible because Cas13 enzymes lack the DNA-cleaving RuvC and HNH domains. As a result, Cas13-based RNA editing systems can prevent genomic off-targets or indels brought on by non-homologous end joining (NHEJ) and are likely to be reversible. According to Abudayyeh et al. (2019), CRISPR-Cas13a, also known as C2c2, is a single-effector Class II, Type VI RNA-guided RNA editing system. It has been demonstrated to successfully cleave molecules of single-stranded RNA (ssRNA). Cas13a’s adaptability is further increased by its ideal proto-spacer length (crRNA) and particular PFS preferences. Another notable aspect of the CRISPR-Cas13a system is multiplex editing. It can take a CRISPR array with several guides and process and produce mature crRNA from it, enabling the simultaneous targeting of many sequences from various genes. Both the CRISPR-Cas9 and CRISPR-Cas13a systems in plants have successfully undergone multiplex editing (Aman et al., 2018). Additionally, Cas13a shows promise in the sensitive detection of genetic molecules, which might be extremely helpful in the early identification of epidemics and the diagnosis of diseases. Cas13a may be reprogrammed to allow target RNA to start a collateral action, enabling it to identify particular RNA molecules *in vivo* and *in vitro* with great sensitivity and specificity. It is a possible candidate for the development of a lateral flow assay for the early observation of RNA viruses because of its superiority over presently used techniques like RT-PCR and qPCR in the capacity to identify a single copy of RNA. In conclusion, Cas13 enzymes have brought about a new era of RNA editing and detection, providing special benefits and fascinating opportunities for study in a variety of domains, including plant genetic engineering. These advancements have the potential to completely alter scientific research and open the door to discoveries.

## Base editing

Base editors are a ground-breaking addition to the toolkit for genome engineering, providing a quick and accurate way to substitute nucleotides without the necessity for donor templates or double-stranded breaks (DSBs) (Tiwari and Ghag, 2024). Cytidine and adenine deaminase-based base editors have been effectively created and applied for base editing in both plants and animals during the past few years. Constant improvements have increased the capabilities of CBEs and ABEs, making them more appropriate for agricultural plants. Examples of these improvements include reducing the catalytic window and using enhanced Cas9 variations. Base editors are chimeric proteins that demethylate cytosine or adenine bases in the genome; they consist of a DNA targeting module and a catalytic domain (Komor et al., 2016; Gaudelli et al., 2017). Besides, a catalytically inactive Cas9 endonuclease (dCas9) or a Cas9 nickase directed by a single-guide RNA (sgRNA) molecule can be used as the DNA targeting module. When it comes to dCas9, particular mutations (Asp10Ala and His840Ala) render its nuclease action inactive but leave it still able to bind DNA. An “R-loop,” or short length of unpaired DNA, is produced when dCas9-sgRNA binds to the target DNA. The tethered deaminase can change cytosines or adenines inside this tiny single-stranded area of roughly 5-8 nucleotides. Base editors can reduce the number of indels by introducing single-base alterations or replacements without triggering DSBs. Cytosine base editors (CBEs) and adenine base editors (ABEs) are the two primary categories of DNA base editors. While ABEs help with the translation of A-to-G or T-to-C, CBEs can convert C to T or G to A. Future crop development might be sped up with the use of these exact base editing tools, which have enormous promise for precision breeding in model plants and crops (Mishra et al., 2020). Despite the bright future, base editing technology is still in its inception. To increase efficiency and specificity, additional development and an extension of its editing scope are required. It could be conceivable to continually improve base editors and look at novel mutant forms of cytidine deaminase to boost DNA selectivity and lessen off-target effects. With these developments, the area of plant genetic engineering will undergo a revolution as extremely accurate base editors are extensively used in crop enhancement initiatives.

CBEs are effective tools that catalyse the transformation of cytosines into thymines. The cytidine deaminase enzyme, which separates an amino group from cytosine and produces uracil as a consequence, is responsible for this conversion. Following the correction of the uracil-guanine (U-G) mismatch via DNA repair pathways, uracil-adenine (U-A) base pairs are created. As a result, thymine is added to the freshly synthesized DNA strand, changing C-G into T-A. David Liu and his group at Harvard University in the United States created the first-generation CBE, sometimes referred to as BE1, in 2016. It had a 16-amino acid XTEN linker that connected the rat-derived APOBEC1 cytidine deaminase enzyme to a catalytically inactive Cas9 (dCas9) (Komor et al., 2016). A balance between the two proteins is maintained by the XTEN linker. The APOBEC family of cytidine deaminases, which function on single-stranded DNA and RNA as substrates, are naturally occurring enzymes that protect vertebrates against viral infections. The recurrent removal of uracil by uracil DNA glycosylase (UDG), which led to poor editing ability, was one of BE1’s key drawbacks. The second-generation base editor BE2 (APOBEC-XTEN-dCas9-UGI) was created to get around this restriction. It entailed incorporating a uracil DNA glycosylase inhibitor (UGI) into the DNA targeting module’s C-terminus, which increased editing effectiveness (Komor et al., 2016). Multiple cytosines (Cs) present inside the catalytic window provide a problem for CBEs, potentially leading to off-target action and the changing of non-target cytosines to uracil. Several BE3 variations were created using various Cas9 variants and non-canonical PAM sequences to overcome this problem. For instance, SpCas9 variants that target the NGAN, NGAG, NGCG, and NRT PAMs, respectively, such as VQR-BE3, EQR-BE3, VRER-BE3, and SaKKH-BE3, have demonstrated a 2.5-fold improvement in editing ability (Kim et al., 2017). The accuracy and efficacy of base editing have considerably enlarged thanks to these developments in CBE technology, creating new opportunities for precise genome engineering in a variety of taxa, along with plants and mammals.

Adenine bases may now be precisely modified in a programmed manner thanks to ABEs, which is an exciting development in genome editing technology (Gaudeli et al., 2017). Adenine DNA deaminases are not found in nature, in contrast to cytidine deaminases, which are naturally occurring enzymes. David Liu and his colleagues used *Escherichia coli* TadA (*E. coli* TadA) and carried out substantial protein engineering and guided advancement to create ABEs. With similarity to the APOBEC enzyme family, *E. coli* TadA is a tRNA adenine deaminase that transforms adenine to inosine in the single-stranded anticodon loop of tRNA Arg. By combining *E. coli* TadA with a catalytically imperfect CRISPR/Cas9 mutant, the first-generation adenine base editors, or ABEs, were produced (Gaudeli et al., 2017). ABE7.7, ABE7.8, and ABE7.9 from the newly created family of ABEs showed significant activity as adenine base editors and compatibility with a wider variety of target sequences. The seventh generation of ABE, also known as ABE7.10, was evolved later and greatly enhanced the effectiveness and product purity for converting A.T. to G.C. in a variety of target sequences. By permitting all four transitions—C to T, A to G, T to C, and G to A—in a programmable way, ABEs have considerably increased the range of base editing. Recently, base editor rBE5 was used in rice to target Pi-d2gene for modulating defense against rice blast fungus (Ran et al., 2018). ABEs are excellent instruments for precisely introducing point mutations due to their enhanced adaptability and efficiency. The use of ABEs for targeted base editing creates new avenues for precisely targeting certain genetic mutations and creating treatments.

## Prime editing and RNA base editors (ADAR)

To get over the constraints of HDR-mediated editing, prime editing represents a substantial leap in genome editing technology (Tiwari and Ghag, 2024). It can carry out a variety of modifications, including 1–80 bp deletions, 1–44 bp insertions, and all base substitutions in human cells (Anzalone et al., 2019). The primary editing method is built on a special guide RNA known as peg RNA that has a reverse transcriptase (RT) template with the needed alteration and a primer binding site (PBS). On the nicked strand of genomic DNA, the PBS sequence hybridizes with the complementary target sequence. The target DNA sequence is subsequently precisely edited by the RT, which uses this hybridized template to facilitate the synthesis of the necessary modifications. The RESCUE (RNA Editing for Specific C-to-U Exchange) system for RNA editing is another cutting-edge technique. The RESCUE system was created to address some inherent shortcomings of natural cytidine deaminases for RNA editing, and it has shown up to 42% editing efficiency in endogenous transcripts. But around the targeted nucleotide, RESCUE showed off-target edits, including C-to-U and A-to-I. Guanine mismatches were added to the guide RNA across from the off-target edits to lessen these off-target effects. This change enabled Rescue to make fewer local, off-target C-to-U edits. In addition, rational mutagenesis was carried out, resulting in the discovery of the RESCUE-S variant (Abudayyeh et al., 2019). This variant significantly reduced off-target C-to-U edits by about 45% and off-target A-to-I edits by about 94%, similar to the original RESCUE system. These developments in prime editing and the RESCUE system have enormous potential to broaden the application of precise genome editing and solve the problems brought on by off-target effects. These technologies can change the field of genetic engineering and open the door to numerous uses in research, biotechnology, and therapeutic interventions with additional optimization and refinement.

Feng Zhang and his team were the ones who first created RNA base editors, also known as ADAR (adenosine deaminase acting on RNA) base editors. Adenosine-to-inosine transformation in mammalian cells was made possible by the use of the naturally occurring ADAR enzyme and a catalytically inactive form of the Cas13 protein (dCas13) (Cox et al., 2017). Cas13 is a type VI RNA-guided RNase that is associated with CRISPR and can bind to RNA molecules. The *Prevotella* sp. Cas13b ortholog, one of the tested Cas13 enzymes, showed greater effectiveness and specificity in RNA binding and knockout operations. By catalysing the hydrolytic deamination of adenosine to inosine and thereby enabling precise base editing in RNA, the ADAR family of enzymes is in charge of the endogenous editing of transcripts (Nishikura, 2010). Targeted RNA editing of transcripts is possible with the help of this system, noted as RNA Editing for Programmable A to I Replacement (REPAIR). With even greater specificity compared to earlier RNA editing platforms, REPAIRv2—an improved version of the original REPAIR—was later produced (Stafforst et al., 2012). According to Ferreira et al. (2010), the REPAIR system has proven to be very successful at simulating protective alleles that protect against a variety of autoimmune diseases. It provides a favourable RNA editing platform with numerous uses in biotechnology, therapeutic interventions, and research. With the ability to precisely edit RNA, new avenues for treating genetic disorders and improving our knowledge of how genes work and are regulated are now possible. RNA-base editing holds great potential for a variety of applications as the technology develops and improves.

## Genome editing enhances host plant resistance

The use of disease-resistant cultivars is the most efficient and ecologically friendly method of controlling plant diseases. Most plant pathogens are known to make use of dominantly inherited genes, so-called S genes, in order to successfully develop disease and proliferate in their host. However, genetic disruption of these genes has proven to be one of the most effective methods for mitigating plant diseases and inducing long-term disease resistance. Interestingly, novel genome-editing methods provide prospects for controlling bacterial, viral, and fungal infections, as well as implementing pathogen resistance in plants (Karmakar et al., 2022). Among genome editing tools, CRISPR/Cas9 has been regarded as a successful technique due to its versatility, lower cost, ease of design and implementation, and increased success rate (Saraswat et al., 2024).

## Genome editing for improving disease resistance against bacterial diseases

The rapid rate of plant pathogenic bacteria’s multiplication makes it difficult to control infestations of these diseases using conventional methods. However, specific genomic modifications have been successfully introduced into the sensitivity genes of host crop plants using genome editing technology. For instance, CRISPR-Cas9 technology was used to make resistance against the pathogen in the case of citrus canker, a serious bacterial disease caused by *Xanthomonas citri sp. Citri* (Table 2). It was discovered that the vulnerability gene Lateral Organ Boundaries 1 (CsLOB1) is essential for the pathogen’s expansion and the emergence of symptoms. The genome-edited citrus plants displayed protection from citrus canker disease without interfering with the gene’s typical developmental functions by targeting the effector binding element (EBEPthA4) of the CsLOB1 promoter, which is bound by the transcription activator-like effectors (TALEs) (Peng et al., 2017). Similar to bacterial blight, which has a devastating effect on rice production in Asia and Africa, *X. oryzae pv. oryzae* is the pathogen responsible. The pathogen secretes TALEs that bind to particular promoter sequences and cause the expression of the genes *SWEET11, SWEET13*, and *SWEET14,* sucrose-specific transporter genes that are crucial for disease susceptibility. Rice plants with disease resistance were produced by using TALEN-mediated genome editing to reduce the effector-induced transcriptional activation of the susceptibility gene OsSWEET14 (Li et al., 2012). CRISPR/Cas 9 was used to target the gene OsSWEET11 in rice to produce a bacterial leaf blight-resistant line (Roviqowati et al., 2024). It has been demonstrated that the CRISPR-Cas9 genome editing technology is a useful tool for causing mutations in particular genes to confer disease resistance in crops against dissimilar viral pathogens.

**TABLE 2:** Application of genome editing for improving disease resistance in plants by targeting different genes.

Disease	Pathogen	Nuclease	Target gene	Mutation	Host plant	References
DNA viral disease	Beet severe curly top virus (BSCTV)	AZP	Replication origin	n.d	Arabidopsis	Sera (2005)
DNA viral disease	Tomato yellow leaf curl China virus (TYLCCNV)/Tobacco curly shoot virus (TbCSV)	ZFN	Rep	n.d	Tobacco	Chen et al. (2014)
DNA viral disease	Tomato yellow leaf curl China virus (TYLCCNV)/Tobacco curly shoot virus (TbCSV)/Tomato leaf curl Yunnan virus (TLCYnV)	TALE	Rep	n.d	Tobacco	Cheng et al. (2015)
Rice bacterial blight	*X. oryzae* pv*.oryzae*	TALEN	*OsSWEET14*/promoter	In-dels	Rice	Li et al. (2012)
Powdery mildew	*Blumeria graminis* f. sp *.tritici*	TALEN	*TaMLO*/exon	In-dels	Wheat	Wang et al. (2014)
Late blight	*Phytopthora infestans*	CRISPR/Cas9	*StPM1*	n.d	Potato	Bi et al. (2024)
Rice bacterial blight	*X. oryzae* pv*.oryzae*	CRISPR/Cas9	OsSWEET13/exon	deletions	Rice	Zhou et al. (2015)
Rice blast	*M. oryzae*	CRISPR/Cas9	*OsERF922/exon*	In-dels	Rice	Wang et al. (2016)
Citrus canker	*X. citri subsp.citri*	CRISPR/Cas9	*CsLO1/exon*	In-dels	Citrus	Jia et al. (2017)
Powdery mildew	*B. graminis* f. sp *.tritici*	CRISPR/Cas9	*TaEDR1*/exon	In-dels	Wheat	Zhang et al. (2017)
Citrus canker	*X. citri subsp.citri*	CRISPR/Cas9	*CsLOB1/promoter*	deletions	Citrus	Peng et al. (2017)
Powdery mildew	*Oidium neolycopersici*	CRISPR/Cas9	*SIMlo1*/exon	deletions	Tomato	Nekrasov et al. (2017)
DNA viral disease	Beet severe curly top virus (BSCTV)	CRISPR/Cas9	IR, CP, Rep	In-dels	Tobacco/Arabidopsis	Ji et al. (2015)
RNA viral disease	Cucumber vein yellowing virus (Ipomovirus) (CVYV), Zucchini yellow mosaic virus (ZYMV), Papaya ringspot virus watermelon strain (PRSV-W)	CRISPR/Cas9	*elF4E/exon*	deletions	Cucumber	Chandrasekaran et al. (2016)
RNA viral disease	Cucumber mosaic virus (CMV)	CRISPR/Cas9	*ORF1a, ORFCP* and 3′-UTR	No cleavege	Tobacco/Arabidopsis	Zhang et al. (2018)
RNA viral disease	Turnip mosaic virus (TuMV)	CRISPR/Cas13a	*GFP,Hv-Pro and CP*	n.d.	Tobacco	Aman et al. (2018)

## Genome editing for improving disease resistance against viral diseases

CRISPR-Cas technology is used in two main ways to build virus resistance in crops. In the first strategy, viral disease resistance is attained by carefully destroying the viral pathogen’s genome. For instance, the foundation of the sgRNA-Cas9 construct targeting the viral genome followed the development of plants resistant to the Geminivirus in Arabidopsis and *N. benthamiana* (Ji et al., 2015). Alike findings were obtained in *N. benthamiana* by designing sgRNAs against the coding and non-coding sequences of the tomato yellow leaf curl virus (TYLCV), which resulted in transgenic plants with decreased viral DNA accumulation and TYLCV resistance (Ali et al., 2015). The second strategy entails editing host genes that encode susceptibility factors (S genes) to make crops that are resistant to viruses. Genome editing was used to target the eIF4E gene in the cucumber (L.), which is a susceptibility factor. As a result, transformed plants have resistance to various phytoviruses and ipomoviruses (Chandrasekaran et al., 2016). Adopting the CRISPR-Cas9 system, the wheat dwarf virus (WDV), a member of the Geminiviridae family, was targeted at four sites in its genome to create stable WDV-resistant wheat lines with few off-target effects (Kis et al., 2019). Additionally, the CRISPR-Cas9 innovation has been profitably used to combat other viruses like the tomato yellow leaf curl virus (TYLCV), bean yellow dwarf virus, and beet severe curly top virus (BSCTV) (Ali et al., 2015; Baltes et al., 2015; Ji et al., 2015). Along with these methods, genome editing innovations have made it possible to introduce genetic variations that are naturally present in wild cultivars, particularly recessive traits that are primarily governed by genes like the translation initiation factor 4 gamma gene (eIF4G), which confers disease resistance. To confer protection against the Rice tungro spherical virus (RTSV), Macovei et al. (2018) modified the eIF4G gene of the IR64 rice variety using the CRISPR-Cas9 tool. For breeding and spreading hybrids of Musa species, the presence of endogenous banana streak virus (eBSV) in the B genome poses a significant barrier. To inactivate eBSV, CRISPR-Cas9 gene editing was used. This resulted in targeted mutations in all three open reading frames (ORFs) and the successful removal of integrated dsDNA from BSV from the banana genome. This innovation has made it easier to improve the Plantain B genome germplasm, according to Tripathi et al. (2019). The CRISPR-Cas9 system has also been profitably used to confer resistance against RNA viruses like the cucumber mosaic virus (CMV) and tobacco mosaic virus (TMV), in addition to DNA viruses. Reduced viral infection symptoms and viral RNA accumulation in the genome-edited plants were the results of targeting CMV and TMV with Francisella novicida Cas9 (FnCas9) in combination with particular sgRNAs (Zhang et al., 2018). CRISPR-Cas9 was used to target single-stranded DNA-A of the AV1 and AC1 genes in the Mung bean yellow mosaic virus to enhance resistance in tobacco against this virus (Talakayala et al., 2024). RNA viruses that harm plant cells can be targeted using the CRISPR-Cas13 system in addition to the CRISPR-Cas9 system. When different Cas13 systems were characterized by Mahas et al. (2019), they discovered that the LwaCas13a, PspCas13b and CasRx variants had the best defense against RNA viruses. CasRx is a highly targeted and programmable tool for transcriptome engineering because it has demonstrated high specificity for its target RNA viruses and minimal off-target effects in plant cells.

## Role of genome editing for enhancing resistance against fungal diseases

Plants that have been genetically changed modified the CRISPR-Cas9 system are significantly more resistant to fungal diseases. To increase protection against the fungus *Magnaporthe oryzae*, Wang et al. (2016) targeted ethylene-responsive factors (ERF) in rice. Similar to this, in hexaploid wheat, heritable mutations in the *MILDEW-RESISTANCE LOCUS (MLO)* genes were introduced using TALEN and CRISPR-Cas9 technologies, providing notable protection against powdery mildew disease (Wang et al., 2014). Additionally, employing genome deletion using CRISPR-Cas9 technology, Nekrasov et al. (2017) created the tomato variety “Tomelo,” which is resistant to the powdery mildew fungus. This variety was created quickly, and it is non-transgenic and free of foreign DNA sequences. To create a cultivar of potatoes that is resistant to late blight disease, Kieu et al. (2021) discovered potential S-genes and underwent genome editing. These susceptibility genes’ (S-genes) absence results in increased resistance to the late blight pathogen. Knocking out *RECEPTOR-LIKE KINASE 902 (RLK902)* by CRISPR-Cas9 in *Brassica napus* displayed a higher level of resistance against *Sclerotinia sclerotiorum* and *B. cinerea* (Zhao et al., 2024). Overall, the use of CRISPR-Cas technologies to build disease resistance in different crops has notable potential to increase agricultural output and fight plant diseases. These examples demonstrate how the evolution of disease-resistant crop plants is now possible thanks to genome editing technology that targets both host genes and pathogenic organisms. Genome editing’s capacity to make precise and targeted changes holds great potential for reducing the financial losses brought on by plant diseases and raising agricultural production worldwide.

## Success rate and limitations of genome editing mediated disease resistance against pathogens

The success rate of genome editing in conferring disease resistance against pathogens in plants varies depending on several factors, including the target crop, the specific pathogen, the genetic basis of resistance, and the efficiency of the editing technique employed. Some crops and pathogens have been more amenable to successful genome editing-mediated disease resistance than others. For example, in crops like rice, wheat, and tomato, researchers have achieved significant success in enhancing resistance against various pathogens such as rice blast fungus, wheat powdery mildew, and bacterial wilt in tomatoes through genome editing (Wang et al., 2014; Nekrasov et al., 2017; Li et al., 2018). However, success rates can vary when targeting different pathogens within the same crop species due to variations in the genetic basis of resistance and the complexity of the pathogen’s interaction with the plant. The choice of genome editing technique can also influence the success rate. CRISPR/Cas-based systems have emerged as powerful tools for precise genome editing in plants due to their efficiency, versatility, and relatively low cost compared to older techniques like zinc finger nucleases (ZFNs) and transcription activator-like effector nucleases (TALENs) (Wang et al., 2014). Continuous improvements in CRISPR/Cas systems, such as the development of high-fidelity Cas variants and delivery methods, have further enhanced the success rates of genome editing-mediated disease resistance. The success of genome editing-mediated disease resistance depends on identifying and targeting genes or genetic elements that confer resistance to the pathogen. In some cases, single genes may confer strong resistance, making them ideal targets for editing. However, in other instances, resistance may be governed by multiple genes or quantitative trait loci (QTLs), requiring more complex editing strategies such as gene stacking or modification of regulatory elements (Zaidi et al., 2018). Efficient delivery of genome editing tools into plant cells remains a technical challenge, particularly for crops with recalcitrant or complex genomes, limiting the practical application of these techniques (Mushtaq et al., 2019).

Genome editing holds promise for bolstering plant disease resistance, but several limitations and barriers hinder its widespread application such as off-target effects, regulatory hurdles, Complex genetic interactions and ethical concerns. For example, genome editing techniques such as CRISPR/Cas9 may inadvertently incorporate mutations in unintended regions of the genome, potentially leading to unforeseen consequences (Chen et al., 2022). The regulatory frameworks governing genome-edited crops vary globally and can pose significant barriers to the commercialization and adoption of disease-resistant plants (Entine et al., 2021). The genetic basis of disease resistance in plants is often multifaceted, involving numerous genes and regulatory elements, making it challenging to engineer robust resistance using genome editing alone (Mushtaq et al., 2019). Manipulating the genomes of plants raises ethical questions related to environmental impact, biodiversity, and unintended consequences, necessitating careful consideration and public engagement (Chen et al., 2022).

## Conclusion and future outlook

The growing global demand for food, coupled with the challenges posed by climate change, has necessitated advancements in crop resilience and yield improvement strategies. Conventional plant breeding methods have been relied upon for crop amelioration, but the development of genome editing has provided a more precise and targeted approach to modifying crop plants and overcomes the limitations of conventional resistance breeding. Genome editing techniques, such as ZFNs, TALENs, and CRISPR-Cas9, have revolutionized molecular biology and crop sciences, enabling functional genomics and the enhancement of agricultural traits. The application of genome editing tools has demonstrated remarkable efficacy in enhancing crop immunity against various phytopathogens by modifying host disease susceptibility genes. The CRISPR-Cas system, in particular, is well-suited for multiplexing, allowing the targeting of multiple genes to evolve broad-spectrum resistance. Moreover, genome editing offers valuable insights into the molecular mechanisms of pathogenesis and host resistance, opening new avenues for disease diagnosis in plants. Genome editing methods are unparalleled in precision, allowing targeted changes at the gene, regulatory, and nucleotide levels. Genome editing technology can be used to leverage pathogen effector target sequences to develop novel sources of resistance in key agricultural crops, thereby reducing overreliance on pesticide applications and minimizing the necessity for genetically modified (GM) crops. Crops derived from genome editing with minor genomic modifications are virtually indistinguishable from their traditional counterparts, posing negligible risks to the environment, society, economy, and human health. Many countries, as well as India, have de-regulated genome-edited crop varieties that lack foreign DNA, fostering their adoption in agriculture. To further advance genome editing technology, research is needed to explore PAM-independent CRISPR applications and develop strategies to avoid and detect off-target effects more effectively. Additionally, efforts to develop smaller editing tools will enhance their delivery and editing efficiency in plant cells. As we move forward, the advantage of genome editing technologies will be noticed through the commercialization of disease-resistant, climate-resilient, and superior-quality crops. These advancements will not only strengthen global food security but also foster sustainable agricultural practices in the face of evolving environmental challenges.

Despite the challenges and variability in success rates, the future outlook for genome editing-mediated disease resistance in plants is promising. Continued advancements in genome editing technologies, coupled with increasing knowledge of plant-pathogen interactions and genetic mechanisms underlying resistance, are expected to improve success rates and expand the range of target crops and pathogens. Furthermore, ongoing research efforts are focused on developing innovative strategies to enhance the efficacy and durability of edited resistance traits, such as combining genome editing with other breeding approaches like marker-assisted selection and RNA interference (RNAi), as well as leveraging synthetic biology tools to engineer novel resistance mechanisms. Overall, genome editing holds immense potential for revolutionizing plant disease resistance breeding by providing precise and sustainable solutions to combat pathogens, thereby contributing to global food security and agricultural sustainability.
